# *In vivo* imaging of the Muc5b gel-forming mucin

**DOI:** 10.1038/srep44591

**Published:** 2017-03-15

**Authors:** Céline Portal, Valérie Gouyer, Mylène Magnien, Ségolène Plet, Frédéric Gottrand, Jean-Luc Desseyn

**Affiliations:** 1 LIRIC UMR 995, Univ. Lille, Inserm, CHU Lille, F–59000 Lille, France

## Abstract

Gel-forming mucins are macromolecules produced by goblet cells and responsible for the mucus gel formation. Changes in goblet cell density and in gel-forming mucin production have emerged as sensitive indicators for mucosal diseases. A Muc5b-GFP tagged reporter mouse was used to assess Muc5b production in mouse tissues by immunofluorescence microscopy and fluorescent activity using stereromicroscopy and probe-based confocal laser endomicroscopy. Muc5b production was followed longitudinally by recording the fluorescent activity in vagina and in embryonic lung explants under stimulation by interleukin 13. We show that the GFP is easily visualized in the mouse adult ear, nose, trachea, gallbladder, and cervix. Live Muc5b is also easily monitored in the nasal cavity, trachea and vagina where its production varies during the estrus cycle with a peak at the proestrus phase and in pregnant mice. Explant culture of reporter mouse embryonic whole lung shows that interleukin 13 stimulates Muc5b production. The transgenic Muc5b-GFP mouse is unique and suitable to study the mechanisms that regulate Muc5b production/secretion and mucous cell differentiation by live imaging and can be applied to test drug efficacy in mucosal disease models.

Mucus hydrogels coat mucosal surfaces and protect them from chemical, enzymatic and mechanical damages. Main functions of mucus gels include lubrication and hydration of epithelial surfaces and clearing dust and bacteria. Gel-forming mucins are large secreted polymeric molecules of several MDa that form the matrix of mucus gels. They are responsible for the structure, adhesion, and rheological properties of the gel^1^,^2^. Mucins carry many extended *O*-glycosylated chains making them difficult to study. They are synthesized and secreted by specialized cells located at the mucosal surface or in submucosal glands^3^. Abnormal mucin production is a feature of many mucosal diseases where the mucous cell density reflects the disease state making mucins informative molecular markers to quantify disease progression and regeneration and repair upon pharmacological treatments^4^,^5^,^6^,^7^.

Five gel-forming mucins have been identified in humans and named MUC2, MUC5AC, MUC5B, MUC6, and MUC19. These mucins are highly conserved between species and named Muc2, Muc5ac, Muc5b, Muc6, and Muc19 in mice^8^,^9^. MUC5B has been reported to be dysregulated in many mucosal disorders (Table 1) but less is known of its mouse counterpart. Moreover, only sparse information on *in vivo* mucin gene regulation is available. We recently created a genetically modified mouse strain by knock-in insertion of a monomeric enhanced green fluorescent protein (GFP) sequence as a reporter just downstream of the last amino acid of the gel-forming mucin Muc5b (Fig. 1a). The reporter Muc5b-GFP mouse was used here to extend our knowledge on the expression pattern of Muc5b by immunofluorescence microscopy, fluorescent activity and by providing noninvasive live imaging of the lumen of the trachea, nose and vagina. Our data suggest that Muc5b production in the cervix of living mice is hormonally regulated and we bring a proof of concept that the transgenic reporter mouse allows to monitor Muc5b production upon drug administration. To this end, we assessed Muc5b production in embryonic whole lung explant culture stimulated by recombinant interleukin 13 (rIL13).

## Materials and Methods

### Mice

A transgenic reporter mouse was created by homologous recombination where the full amino acid sequence of the gel-forming mucin Muc5b was tagged with a monomeric enhanced GFP peptide sequence (manuscript submitted). Heterozygous (Muc5b^gfp/+^) and homozygous (Muc5b^gfp/gfp^) Muc5b-GFP mice which are healthy and fertile were used throughout this study using their wild-type (WT) littermates as a negative control for fluorescence activity. Mouse genotypes were determined by PCR using the two primers 5′-GTCAGGCATCTCATGCTCACAAAAGC-3′ and 5′-AGGATGTAGGGTCCTAGCACCAATGTAGC-3′. For time breeding, females were examined daily for a vaginal plug. The day of the plug was considered embryonic day 0.5 (E0.5). Mice were housed in a specific-pathogen free animal facility. Animal protocols were approved by the Animal Care and Use Committee of the region Nord–Pas de Calais (protocol 1606-2015090217056239) and in accordance with the French Guide for the Care and Use of Laboratory Animals and with the guidelines of the European Union.

### Tissue collection, stereomicroscopy, histology, and immunofluorescence microscopy

Mice were killed and tissues were removed and rinsed in phosphate-buffered saline (PBS) (Gibco BRL, France). Stereomicroscopic pictures were taken using a M205 stereomicroscope (Leica) equipped with a color DFC450c camera (Leica). Histological studies using either periodic acid-Schiff (PAS) or alcian blue (AB)–PAS and immunofluorescence studies using specific primary polyclonal anti-Muc5b antibody^10^ and anti-GFP antibody (Ab290, Abcam, France) were performed as described previously^10^. For immunohistochemistry of the nose and the middle ear, excess soft tissue was removed. The nose and the middle ear were decalcified in 10% EDTA for 6 weeks and in 12% formic acid for 3 weeks, respectively. Histological and immunofluorescence analyses were performed on a Leica DM4000B. High-quality captures in bright field and fluorescence were performed and digitized for the cervix on a Carl Zeiss Axio Scan. Z1 scanner and processed with ZEN software.

### *In vivo* imaging

Live GFP activity was recorded by probe-based confocal laser endomicroscopy (pCLE) using Cellvizio apparatus (Mauna Kea Technologies, Paris, France) as described previously^11^. For the cervix studies, 10–30 sec-recorded movies were acquired. Three independent observers scored the GFP intensity from 1 (almost no GFP spots) to 4 (high content of fluorescent mucus). On the 28 movies, 21 showed an identical score between the three observers and seven a score that differed by one graduation between two observers. The median of the three scores was considered. Identification of the stage of the estrous cycle was performed by analysis of vaginal secretions^12^. The vagina from five transgenic pregnant mice (from E11.5 to E17.5) and a non-transgenic mouse (E15.5) was also observed by pCLE. GFP activity in the vagina was scored by eye independently by three observers unaware of the estrus phase of mice.

### Embryonic lung explant culture

Mouse embryos at gestational stage E12.5 (stage of early lung branching) were removed from homozygous Muc5b-GFP mothers. Embryos were fixed and embedded in paraffin for further immunohistochemical analyses. Whole embryonic lungs were also dissected from the embryos and embryonic whole lung cultures were performed as described elsewhere^13^. Recombinant IL13 (10 ng/mL; SRP4166, Sigma Aldrich, France) was added into the culture medium on day (D) 0. Lung explants were cultured at 37 °C in 5% CO_2_ for up to 11 days. Two sets of embryos were used and pooled for the statistical analysis. Photographs were taken in bright field (50 ms acquisition) and fluorescent mode (10 sec acquisition without binning at maximum light brightness) using a Leica M205 stereomicroscope equipped with a sCMOS ORCA-Flash4.0 camera (Hamamatsu) at x30.3 magnification without a change in depth or focus. The lung area was measured using ImageJ/FIJI^14^ and normalized to the area at D0. Fluorescence activity was quantify using FIJI and expressed as intensity/area and normalized to the fluorescence activity measured at D0.

### Statistical analysis

Statistical analysis of the data was performed using the StatXact v6.0 statistical software (Cytel Studio, Cambridge, MA) for exact nonparametric inference. The inter-observer agreement for cervical GFP activity estimation was calculated using the values of *κ* from the Cohen’s Kappa test. When statistically significant (*P* value < 0.05), its ranges from −1 to 1, and the interpretation is given by the following ranges: bellow 0 no agreement, 0.01 to 0.20 slight agreement, 0.21 to 0.40 fair agreement, 0.41 to 0.60 moderate agreement, 0.61 to 0.80 substantial agreement, and 0.81to 0.99 almost perfect agreement^15^.

## Results

### Muc5b expression pattern

**Figure 1:**
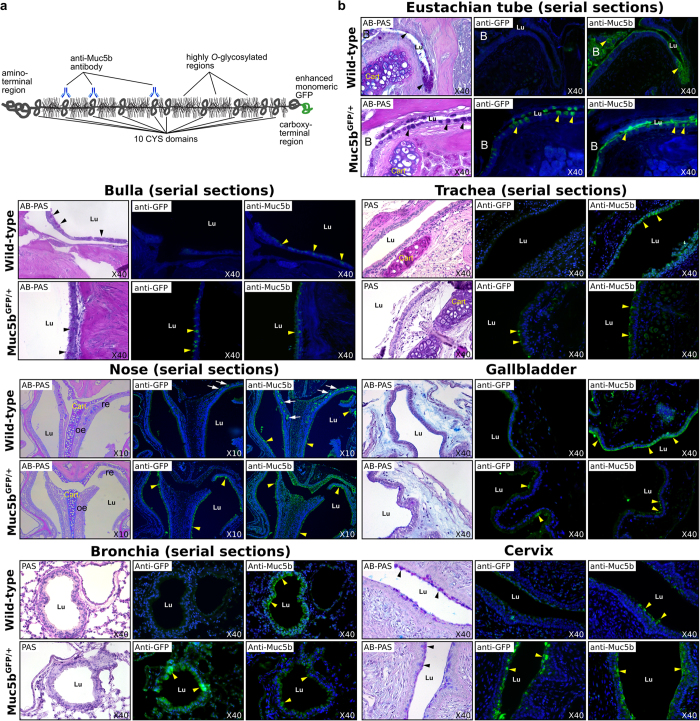
Histological and fluorescence immunohistochemical images of mucosal tissues producing Muc5b. (**a**) Schematic representation of the GFP-tagged Muc5b product. Muc5b forms homopolymers through its amino- and carboxy-terminal regions. These two regions flank the central region which carries many O-glycosylated chains. The central region contains also 10 copies of a CYS domain. The specific anti-Muc5b antibody is directed against a short peptide found in CYS domains #2, 3 and 5^10^. (**b**) Tissues were embedded in paraffin and representative images of n = 3–5 adult mice/genotype (n = 1 mouse/genotype for the nose) are shown. Periodic acid–Schiff (PAS) or alcian blue (AB)–PAS staining of histological sections is shown. Anti-Muc5b (green) and anti-GFP (green) antibodies show the same expression pattern in all studied tissues (eustachian tube, bulla, trachea, nose, gallbladder, bronchia, cervix) with a stronger signal using the anti-GFP antibody. No GFP was found in wild-type mice. B, bulla, black arrow heads outline goblet cells, yellow arrow heads Muc5b material and white arrows Muc5b^+^ glands. Nuclei (blue) were counterstained using Hoechst 33258. Lu, lumen; Cart, cartilage; re, respiratory epithelium; oe, olfactory epithelium.

**Figure 2:**
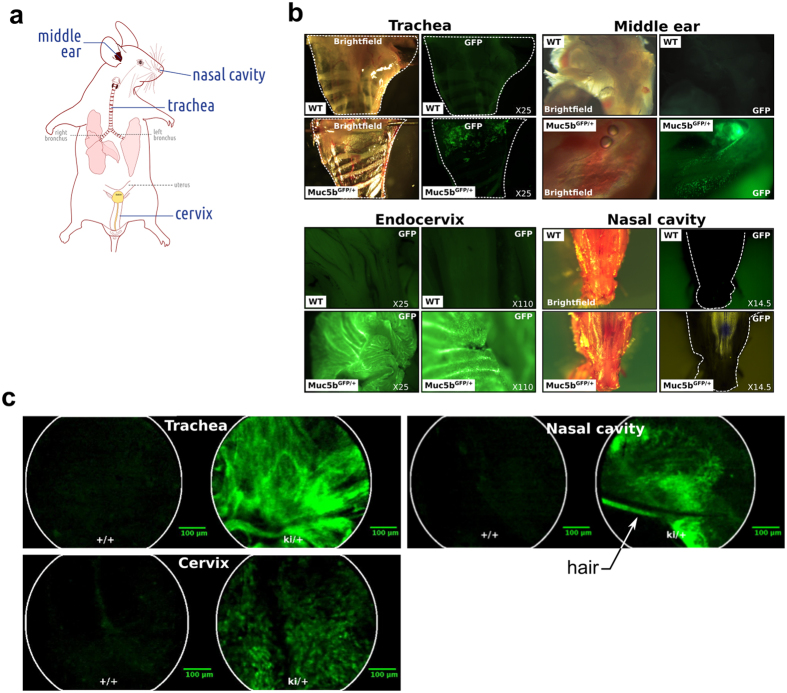
Fluorescent stereo– and endomicroscopy. (**a**) Schematic representation of the tissues that are easily accessible and were studied in this report by probe-based confocal laser endomicroscopy (pCLE). (**b**) Representative pictures of n = 2–4 adult mice/genotype in bright field mode and under GFP excitation by stereomicroscopy of fresh excised tissues from wild-type (WT) and transgenic (Muc5b^GFP/+^) mice. (**c**) Representative single frame of live pCLE imaging (Movies 1, 2, 3) from at least 10 adult mice/genotype for trachea and cervix and 1 adult mouse/genotype for nasal cavity.

To gain more information about the Muc5b production profile in mouse, we performed immunohistochemistry on adult tissues using a commercial antibody directed against the GFP tag and a home-made specific antibody directed against a short peptide found in three CYS domains of Muc5b (Fig. 1a). Both primary antibodies showed that Muc5b is produced by epithelial cells in the eustachian tube, bulla, trachea, nasal cavity turbinate, gallbladder, bronchi (Fig. 1b), and cervix (see below). These data validate our transgenic mouse as a reporter for Muc5b tissue and cell distribution and confirmed the specificity of our anti-Muc5b antibody. We then looked for GFP fluorescent activity using epifluorescence stereomicroscopy in excised tissues with an epithelial cavity producing Muc5b and which is easily accessible to further endomicroscopic studies in living mice (Fig. 2a). An intense signal in comparison to immunohistochemistry was observed in the four tissues studied: trachea, middle ear, cervix, and nasal cavity, while no GFP activity was detected in WT mice (Fig. 2b). We next investigated GFP activity in anesthetized mice using pCLE. Illustrative extracted frames from recorded movies are shown in Fig. 2c. In trachea, GFP activity was mainly visualized as long intermingled threads (Movie 1). In the cervical region (Movie 2) and naso- and/or maxillo-turbinate (Movie 3), GFP activity was recorded as fluorescent spots or as long intermingled threads as in trachea. Due to autofluorescent material in the ear, we did not record convincing specific GFP movies by pCLE. Autofluorescence coming from hairs and possibly from cellulose debris was also sometimes observed in WT and transgenic mice. Nevertheless, these data demonstrate that GFP is easily detectable by pCLE in many tissues in anesthetized mice.

### Muc5b-GFP production seems hormonally regulated in the female reproductive tract

**Figure 3:**
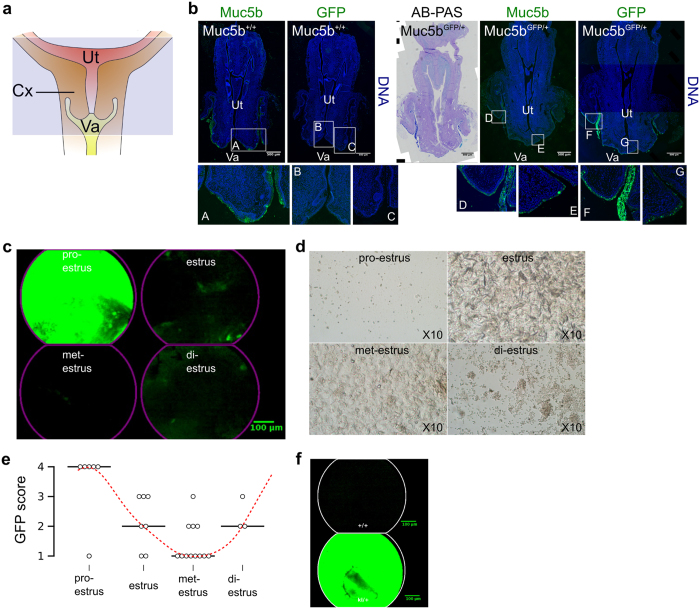
Live GFP activity in the cervix reflects the estrus phase. (**a**) Schematic representation of the mouse cervix (Cx). The mouse uterus (Ut) comprises two horns. Va, vagina. The blue rectangle indicates the area observed by immunofluorescence microscopy. **(b)** Representative serial sections from 2 adult mice/genotype of the cervix in immunofluorescence from wild-type (Muc5b^+/+^) and transgenic Muc5b-GFP (Muc5b^GFP/+^) mice using anti-Muc5b and anti-GFP antibodies. Scale bar, 500 μm. **(c)** GFP activity was recorded by pCLE imaging and one extracted frame from Movie 4 of each estrus phase is depicted here. Scale bar, 100 μm. (**d**) The stage of the estrus cycle in 28 transgenic mice was determined by common vaginal secretion analysis as illustrated. (**e**) GFP intensity was scored from 1 (almost zero) to 4 (high amount of green mucus) by three independent observers showing a perfect match between green mucus production and the estrus cycle (two-sided Jonckheere-Terpstra test, P = 0.015). Medians are shown. N = 6, 7, 12, 3 mice for pro-estrus, estrus, met-estrus and di-estrus, respectively. (**f**) Representative single frame extracted from Movie 5 of the vagina from a wild-type (+/+) and a transgenic pregnant mouse Muc5b^GFP/+^ (ki/+).

We next investigated Muc5b production by immunofluorescence on sections of cervical tissue from WT and Muc5b-GFP mice using both anti-Muc5b and anti-GFP primary antibodies (Fig. 3a and b). No GFP was detected in WT mice and the two antibodies recognized material within or at the cell surface of goblet cells in transgenic mice. This confirmed the specificity of the anti-Muc5b antibody and showed that Muc5b is produced in the mouse cervix. We next followed Muc5b production by pCLE in the vagina of anesthetized mice during the four phases of the estrus cycle (Fig. 3c and d). Representative movies for the four estrus phases showed few green spots at the met-estrus stage, green fluorescent live mucus at the pro-estrus phase and intermediate GFP activity at the estrus and di-estrus phases (Movie 4). Three independent observers scored the GFP intensity from the movies. Cohen’s kappa correlation coefficients and 95% confidence intervals (95%CI) were 0.69 [0.49, 0.90], 0.80 [0.62, 0.98], and 0.80 [0.61, 0.98], respectively (*P* < 0.0001 for all comparisons) indicating substantial inter-observer agreement. The scores demonstrated a perfect relationship between the pro- and met-estrus phases and GFP activity with Muc5b mucin production peaking during pro-estrus (Fig. 3e). We extended our pCLE study to the vagina in transgenic pregnant mice (Movie 5) and observed abundant thick fluorescent mucus (Fig. 3f). These data suggest that Muc5b is hormonally regulated and demonstrate that pCLE is an easy method to follow live fluorescent cervical mucus during the estrus cycle and pregnancy. Our data suggest also that the transgenic Muc5b-GFP mouse represents a good model for live mucus and goblet cell studies in the vagina of mice.

### Fluorescent activity in embryonic lung explant culture is stimulated by rIL13

**Figure 4:**
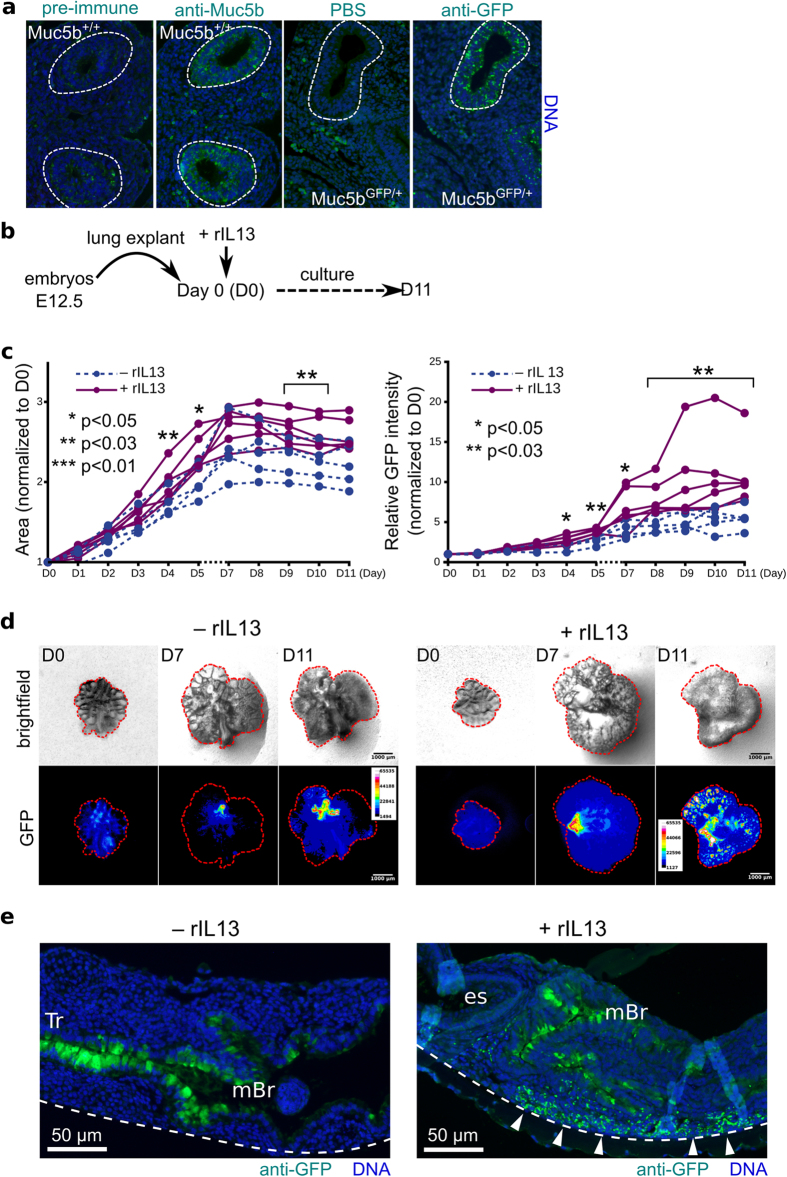
GFP activity in the embryonic whole lung is stimulated by rIL13. (**a**) Representative immunofluorescence staining of an embryonic lung on E12.5 shows similar labeling of the lung bud (dotted line) with anti-Muc5b and anti-GFP antibodies (green). At least 5 pups/genotype were analyzed. Magnification X200. Nuclei (blue) were counterstained with Hoechst 33258. (**b**) Scheme showing the schedule. (**c**) Relative area of embryonic whole lung and relative fluorescent intensity of 10 explant cultures from 10 brother-sisters, five stimulated with rIL13 (+rIL13) and five negative controls (−rIL13). Data were analyzed using the two-sided Wilcoxon–Mann–Whitney test to compare the two groups. (**d**) Representative lung morphology and GFP fluorescent activity of two whole embryonic lung explant cultures at days D0, D7, and D11 without (−rIL13) or with (+rIL13) rlL13 in bright field and pseudo–16 colored images after capture. Beginning at D4 (see panel c), lungs stimulated with rIL13 appeared larger and more fluorescent than control lungs. Scale bar, 1 mm. (**e**) Representative staining sections with anti-GFP antibody (green) of embryonic lung explants after 8 days of culture without (−rIL13) or with (+rIL13) rlL13. Nuclei (blue) were counterstained with Hoechst 33258. The dot line represents the culture filter. White arrow heads show Muc5b production in small bronchi after IL13 stimulation. Tr, trachea; mBr, main bronchus; es, esophagus. Scale bar, 50 μm.

We next chose the embryonic lung as sterile tissue, which has been demonstrated to express Muc5b early during fetal development^16^, to test whether GFP activity of the reporter mucin may be useful in explants under pharmacological tests. Muc5b expression was first studied by immunofluoresence microscopy on embryonic lung tissue sections using both anti-Muc5b and anti-GFP antibodies. Analysis revealed that Muc5b was produced as early as E12.5 using both antibodies (Fig. 4a). We next tested whether rIL13 stimulates Muc5b production in mouse whole embryonic lung explant culture. Whole embryonic lungs were dissected at gestational stage E12.5 and cultured onto filters. Twenty-four hours later, rIL13 was added (day D0) and the lung area and GFP activity were recorded daily by stereomicroscopy until D11 (Fig. 4b). Compared with controls (without rIL13), lungs stimulated by rIL13 exhibited much higher fluorescent activity starting at D4 (Fig. 4c and d). Live fluorescence (Fig. 4d) and immunofluorescence microscopy studies (Fig. 4e) revealed a strong Muc5b production in trachea and in the main bronchus of both unstimulated and rIL13 stimulated embryonic lung cultures. A production of Muc5b was notably observed in distal bronchi explant cultures when stimulated by rIL13 (Fig. 4d and e).

## Discussion

The full pattern of gel-forming mucins expression has not yet been fully determined in mouse, especially that of Muc5b. The two main reasons for this lack of knowledge are the poor availability of specific antibodies and the difficulty in studying mucins because they are very large and heavily glycosylated molecules with glycoforms and natural variation in glycosylation occurring between organs, tissues and cells^3^,^17^. This present work confirms the specificity and reliability of our Muc5b polyclonal antibody for immunohistological studies and provides an important insight into Muc5b expression and production supported by three examples in our study. First, the transgenic reporter mouse showed earlier expression of Muc5b than previously known in mouse embryonic lungs^16^. Second, rIL13 stimulates Muc5b production in whole embryonic lung culture. This is in agreement with studies from other teams which reported that IL13 induces goblet cell differentiation in the airways^6^,^18^ and stimulates proliferation of goblet cells and expression of mucins in primary conjunctival cell cultures^19^. Third, we observed that Muc5b is produced in the cervix and its production seems correlated to gonadal hormones. Although the production of human MUC5B during the menstrual cycle is well documented, nothing was known until our report about Muc5b production in female reproductive mouse tissue. Human MUC5B has been shown to be a major gel-forming mucin in the cervix with a dramatic increase in production just before ovulation^20^,^21^. The exact functions of gel-forming mucins in the cervix are speculative. Their main function is to drive mucus properties which change depending of the estrous cycle. Cervical mucus should permit sperm transport at estrus but must prevent bacterial colonization of the sterile uterus from the colonized vagina. This is supported by the association between preterm birth risk and uterine infection in which cervical mucus permeability appears to be increased^22^,^23^.

Previous studies^10^,^16^ and data presented here give almost the full picture of Muc5b expression/production in adult mice outlining the perfect similarity with humans. This supports the laboratory mouse as a relevant model to study gel-forming mucins and mucous cells in many pathologies. Zebrafish has been recognized as a promising model amongst model organisms to better understand human diseases and to assess drug-induced toxicity in a variety of organs^24^. Five putative gel-forming mucin genes have been identified and genetically GFP-tagged in this species. However, they are not orthologous to the five human gel-forming mucin genes as three seem homologous to either *MUC5AC* or *MUC5B* and two to *MUC2*^25^. Furthermore, the closer evolutionary proximity of mice to humans in many aspects spanning from anatomy to cell biology and physiology makes the mouse a better model for human diseases and gene studies than fish^24^.

Our reporter mouse reveals to be an invaluable tool to monitor live Muc5b expression under its native regulatory elements and live Muc5b production and secretion over time in both explant culture and anesthetized mice. In living mice, this is possible because Muc5b is produced in many accessible epithelial cavities for the pCLE probe and because we used an enhanced GFP variant. Nevertheless, the diameter (300–650 μm), the lack of flexibility and the sharpness of the pCLE fiber we use for epithelial surface imaging are the three main limitations of the technology in small animals. Moreover, small diameter of epithelial cavities in rodents prevents to couple the pCLE probe with standard endoscope. Despite these substantial limitations, pCLE is a very promising technology for mucin visualization in living genetically modified animals. In our study, we sometimes encountered autofluorescence during *in vivo* imaging of hairs (see Movie 3 for example) and likely particles of cellulose origin that can stick on the probe and may interfere with the fluorescence of the GFP tag under the 488 nm laser of pCLE. This major obstacle for automated fluorescent quantification could be overcome using the mCherry tag and the last generation of pCLE apparatus equipped with dual excitation wavelengths at 488 and 660 nm^26^.

Because abnormal mucous cell density reflects tissue damages and remodeling in mucosal diseases^4^,^5^,^6^,^7^, the reporter mouse may help to better understand mechanistic basis of diseases affecting mucous cell density and to test treatment compounds to restore goblet cell density in the numerous mouse models available for mucosal disorders (Table 1). The visualization and quantification of Muc5b expression in explants or in a small cohort of living mice and possibly over time should greatly help the discovery of new effective treatments.

## Additional Information

**How to cite this article:** Portal, C. *et al. In vivo* imaging of the Muc5b gel-forming mucin. *Sci. Rep.* **7**, 44591; doi: 10.1038/srep44591 (2017).

**Publisher's note:** Springer Nature remains neutral with regard to jurisdictional claims in published maps and institutional affiliations.

## Supplementary Material

Supplementary Information

Supplementary video 1

Supplementary video 2

Supplementary video 3

Supplementary video 4

Supplementary video 5

## Figures and Tables

**Table 1:** Human MUC5B and mouse Muc5b in normal and pathological situations.

Human			Mouse		
Normal	Disease	Ref.	Normal	Disease	Ref.
Lung	adenocarcinoma	27	Lung	bacterial infection	28
	IPF^a^	29			
	cystic fibrosis^b^	30,31	cystic fibrosis^c^		10
	panbronchiolitis^b^	32			
Trachea		33	Trachea	asthmatic model	34
Salivary glands		10	Salivary glands		10,17
Nose	chronic rhinosinusitis	35, 36, 37, 38, 39	Nose	allergic rhinitis	40,41
Middle ear & Eustachian tube	otitis^d,e^	35,42, 43, 44	Middle ear & Eustachian tube	otitis	42,45,46
Eye (conjunctiva)			Eye (conjunctiva)		47
Gallbladder^f^	hepatolithiasis	48	Gallbladder		10
Endocervix^g,h^	endometrial tumors	20,49	Endocervix		10
Breast	cancer^i^	50	Mammary tissue	mammary tumors	10
Stomach	gastric cancer	51			

^a^Idiopathic pulmonary fibrosis (sporadic and familial). ^b^Mucous (goblet) cell metaplasia and submucosal gland hyperplasia leading to excessive MUC5B production responsible for airway obstruction. ^c^Increased number of Muc5b-positive cells and Muc5b expression in *Cftr*^−/−^ mice. Presence of mucus plugs in *Cftr*^−/−^ mice experimentally infected with *Pseudomonas aeruginosa*. ^d^Over-secretion of MUC5B in mucoïd media leads to mucus accumulation in the middle ear cavity and causes hearing loss. ^e^In secretory otitis media, the inflammatory-process following bacteria colonization of the middle ear mucosa stimulates mucous cell hyperplasia and metaplasia and up-regulates *MUC5B* gene expression in both the middle ear and eustachian tube. ^f^Abnormal mucin secretion or mucus behavior are thought to contribute to the pathogenesis of gallbladder stone formation, cholecystitis, biliary cancer, and cystic fibrosis-associated gallbladder diseases^52^. ^g^Gel-forming mucin that predominates in the human female endocervical epithelium, peaking prior to midcycle. ^h^MUC5B may exhibit anti-HIV properties^53^ and has been suggested to be a relevant marker to evaluate the safety of candidate microbicides^54^. ^i^In agreement with Valque *et al*.^55^ showing that MCF7 luminal breast tumor cells transfected with recombinant mini-mucin MUC5B showed aggressive behavior of the cells *in vitro* and *in vivo*.
