# Supplementary material for: In vivo imaging of the Muc5b gel-forming mucin
Source: Sci Rep. 2017 Mar 15;7:44591. doi: 10.1038/srep44591 (PMC5353722; doi:10.1038/srep44591)
Supplement: Supplementary Information [file srep44591-s1.pdf]

In vivo imaging of the Muc5b gel-forming mucin

Céline Portal, Valérie Gouyer, Mylène Magnien, Ségolène Plet, Frédéric Gottrand and Jean-Luc Desseyn

Supplementary Data

Movie 1. Representative pCLE in the trachea. Autofluorecent structures (comma shape, first second of movies), which are likely heavily keratinized papillae, are first observed when applying the probe on the tongue of both wild-type (+/+) and Muc5b$^{GFP/+}$ (ki/+) mice before the probe reaches the pharynx and larynx where fluorescent mucus is observed as compact small mucus plugs only for the Muc5b$^{GFP/+}$ mouse. Going deeper, fluorescent mucus is observed in trachea as thick and filamentous structures in the Muc5b$^{GFP/+}$ mouse.

Movie 2. Representative pCLE in the nasal cavity. Fluorescent mucus threads and likely Muc5b-positive mucus cells are observed only for the Muc5b$^{GFP/+}$ (ki/+) mouse and not for the wild-type (+/+) mouse. An autofluorescent hair is also visualized for the Muc5b$^{GFP/+}$ mouse (see Fig. 3c).

Movie 3. Representative pCLE in the cervix. Few autofluorescent particles and light green background are observed for both wild-type (+/+) and Muc5b$^{GFP/+}$ (ki/+) mice at the entrance of the vagina. Afterwards, green dots and threads are visualized for the Muc5b$^{GFP/+}$ mouse but not the for the wild-type mouse.

Movie 4. Representative pCLE in the vagina of Muc5b$^{GFP/+}$ mice **depending on the estrus phase.** A thick fluorescent mucus sticking to the pCLE probe is observed in the vagina in proestrus. In estrus and diestrus, the fluorescent mucus is far less abundant while only autofluorescent particles can be visualized during the metestrus phase.

Movie 5. Representative pCLE in the vagina of pregnant mice. A thick fluorescent mucus sticking to the pCLE probe is observed during pregnancy for the Muc5b$^{GFP/+}$ (ki/+) mouse but not for the wild-type (+/+) mouse.
